# Convolutional neural networks for head and neck tumor segmentation on 7-channel multiparametric MRI: a leave-one-out analysis

**DOI:** 10.1186/s13014-020-01618-z

**Published:** 2020-07-29

**Authors:** Lars Bielak, Nicole Wiedenmann, Arnie Berlin, Nils Henrik Nicolay, Deepa Darshini Gunashekar, Leonard Hägele, Thomas Lottner, Anca-Ligia Grosu, Michael Bock

**Affiliations:** 1 Department of Radiology, Medical Physics, Medical Center University of Freiburg, Faculty of Medicine, University of Freiburg, Freiburg, Germany; 2 German Cancer Consortium (DKTK), Partner Site Freiburg, Freiburg, Germany; 3 Department of Radiation Oncology, Medical Center University of Freiburg, Faculty of Medicine, University of Freiburg, Freiburg, Germany; 4 MathWorks, Inc, Novi, MI USA

**Keywords:** Multi-parametric MRI, Radiation therapy, Automatic tumor segmentation, Convolutional neuronal network

## Abstract

**Background:**

Automatic tumor segmentation based on Convolutional Neural Networks (CNNs) has shown to be a valuable tool in treatment planning and clinical decision making. We investigate the influence of 7 MRI input channels of a CNN with respect to the segmentation performance of head&neck cancer.

**Methods:**

Head&neck cancer patients underwent multi-parametric MRI including T2w, pre- and post-contrast T1w, T2*, perfusion (k_trans_, v_e_) and diffusion (ADC) measurements at 3 time points before and during radiochemotherapy. The 7 different MRI contrasts (input channels) and manually defined gross tumor volumes (primary tumor and lymph node metastases) were used to train CNNs for lesion segmentation. A reference CNN with all input channels was compared to individually trained CNNs where one of the input channels was left out to identify which MRI contrast contributes the most to the tumor segmentation task. A statistical analysis was employed to account for random fluctuations in the segmentation performance.

**Results:**

The CNN segmentation performance scored up to a Dice similarity coefficient (DSC) of 0.65. The network trained without T2* data generally yielded the worst results, with ΔDSC_GTV-T_ = 5.7% for primary tumor and ΔDSC_GTV-Ln_ = 5.8% for lymph node metastases compared to the network containing all input channels. Overall, the ADC input channel showed the least impact on segmentation performance, with ΔDSC_GTV-T_ = 2.4% for primary tumor and ΔDSC_GTV-Ln_ = 2.2% respectively.

**Conclusions:**

We developed a method to reduce overall scan times in MRI protocols by prioritizing those sequences that add most unique information for the task of automatic tumor segmentation. The optimized CNNs could be used to aid in the definition of the GTVs in radiotherapy planning, and the faster imaging protocols will reduce patient scan times which can increase patient compliance.

**Trial registration:**

The trial was registered retrospectively at the German Register for Clinical Studies (DRKS) under register number DRKS00003830 on August 20th, 2015.

## Introduction

Head&neck squamous cell carcinomas (HNSCC) are currently treated with surgery, chemotherapy, radiation therapy or a combination thereof [1, 2] such as primary radio-chemotherapy. In HNSCC radiation therapy, the definition of the gross tumor volume (GTV) on radiological images is an essential step to ensure that the prescribed treatment dose is effectively delivered to the tumor during therapy with only minimal dose spillover into the surrounding healthy tissue [3, 4]. MRI is often used for the GTV target volume, as it provides superior soft tissue contrast compared to CT among other benefits [5, 6]. MRI is often supplemented by positron emission tomography (PET) using novel hypoxia-sensitive tracers such as F-MISO or FAZA to delineate hypoxic subvolumes in the tumor, which require significant dose escalation for an effective tumor treatment.

Manual GTV definition is a tedious and time-consuming procedure which can require up to 2 h per patient, and which is strongly dependent on the training of the executing radio-therapist [7, 8]. To overcome this bias and to accelerate the radiation planning procedure, in recent years automatic tumor segmentation methods have been introduced. These segmentation methods are based on deep learning techniques such as convolutional neural networks (CNN) that have been shown to be highly accurate in the segmentation of various tumor types [9–12], and thus promise to be a valuable tool in assisting experts in clinical decision making.

To reach clinically acceptable and comparable segmentation results, CNNs are usually trained on large tumor image databases which are publicly available [9, 13]. The CNN training with these large amounts of data is very time-consuming as thousands of images must be processed - even on modern graphical processing units (GPU) CNN training can take several days. Once trained, however, the CNNs can make automatic GTV predictions on a single patient data set within seconds thus decreasing the segmentation time by 1–2 orders of magnitude even if small manual corrections must be applied to the automatic segmentation.

In general, deep learning algorithms such as CNNs have shown their potential for modelling complex systems when large amounts of data are available for training [14]. In this context, the volume of data can be twofold: the number of different patients, and the number of different image data sets per patient. The total number of patients is a measure of the data variation that the CNN has seen during training, i.e. the training size. Increasing the training size should make the CNN more robust against variations in the appearance and the localization of the tumor, so that the CNN can better generalize to new patient data. The second factor that determines the available data volume is the number of different image data sets per patient (sometimes called input features or channels) – it encodes the amount of information per patient. For CNNs trained for HNSCC tumor segmentation, the MRI input channels are given by the different contrasts acquired before, during and after therapy. The contrasts in multi-parametric MRI include anatomical and functional contrasts which are acquired in a single imaging session and are, thus, intrinsically co-registered. Unfortunately, the acquisition of multi-parametric MRI data can require imaging times of up to 40 minutes per exam, which can be challenging for HNSCC patients because the images are acquired using a thermoplastic fixation mask covering the complete head-and-neck region that is later used during radiation therapy. During these long exam times patients start to move, and the intrinsic co-registration of the data is compromised, or they even interrupt the exam leading to incomplete data sets. Thus, there is an urgent need to shorten the total exam time by acquiring only those image contrasts that are required for GTV segmentation.

In this study we trained a 3D CNN to for HNSCC tumor segmentation with 7 different MRI contrasts. Based on this, we investigated the relevance of the different MRI input channels with respect to the segmentation performance of the CNN. Therefore, 7 additional CNNs were trained in which one contrast was left out (leave-one-out CNN or LOO-CNN). The outcome of the LOO-CNNs was then compared to the CNN trained on the complete input channels (reference CNN) and the ground truth given by the manually delineated tumor GTV (GTV-T) and lymph node metastases (GTV-Ln) to determine which MRI contrast contributed the least to the segmentation performance.

## Materials and methods

### Clinical data

MRI data from the prospective F-MISO clinical trial was used. In the F-MISO trial correlations between tumor response under radiotherapy and hypoxia in tumor sub-volumes are studied in HNSCC patients. In the time span from 08/2014 to 11/2019, 33 patients have been included, 24 of which underwent 3 T MRI with the complete imaging protocol. Radiation treatment was carried out over the course of 7 weeks in daily 2 Gy fractions to a total dose of 70 Gy. Patients received concomitant chemotherapy with cisplatin (100 mg/m^2^ body surface area) in weeks 1, 4 and 7. The trial was approved in advance by the local Independent Ethics Committee (reference no. 479/12) and was carried out in accordance with the Declaration of Helsinki (revised version of 2015). The trial was registered with the German Clinical Trial Register (DRKS00003830). All 24 patients received multiparametric MRI before treatment (week 0), and at week 2 and 5 during radiation therapy. MRI was performed on a clinical 3 T whole body MR system (Siemens Tim Trio, Erlangen, Germany). During imaging the patients were fixated on the MR patient table with the same mask system that was later used for radiation therapy. As the mask did not allow for the usage of the MR system’s head coil, image data were acquired with a flexible anterior 4-channel array coil and a 2-channel flexible coil in combination with the integrated spine array. The details of the F-MISO MR protocol are listed in Table 1. In general, the protocol consisted of Fast Spin Echo with T1 and T2 weighting, echo planar imaging (EPI) with diffusion weighting, a multiecho gradient echo sequence for T2* calculation, a dynamic 3D gradient echo sequence for dynamic contrast enhanced (DCE) imaging, and a final T1-weighted gradient echo with fat-water separation (Dixon technique). From this data, quantitative apparent diffusion coefficient (ADC) and T2* maps were calculated by voxel-vise exponential fitting, and maps of the perfusion parameters k_trans_ and v_e_ were determined using the Tofts model [15].

**Table 1:** Sequence parameters of the MRI protocol

Sequence	TE [ms]	TR [ms]	Resolution [mm^3^]	Comments / Other
T1 fast Spin Echo	11	504	0.7 × 0.7 × 4	
T2 fast Spin Echo	100	5000	0.7 × 0.7 × 4	
Multi-Echo GRE	5–33	600	1.1 × 1.1 × 3	*n*_Echoes_ = 12, reconstructed map: T2*
DWI	51	2510	2 × 2 × 3	*b* = {50,400,800} s/mm^2^, reconstructed map: ADC
Dynamic T1w Perfusion Measurement	1.56	4.65	1.4 × 1.4 × 3	*n*_Timepoints_ = 36, reconstructed maps: *k*_*trans*_*, v*_*e*_
T1 VIBE Dixon	2.45	8.67	0.45 × 0.45 × 2	Post contrast. Water image used.

**Fig. 1:**
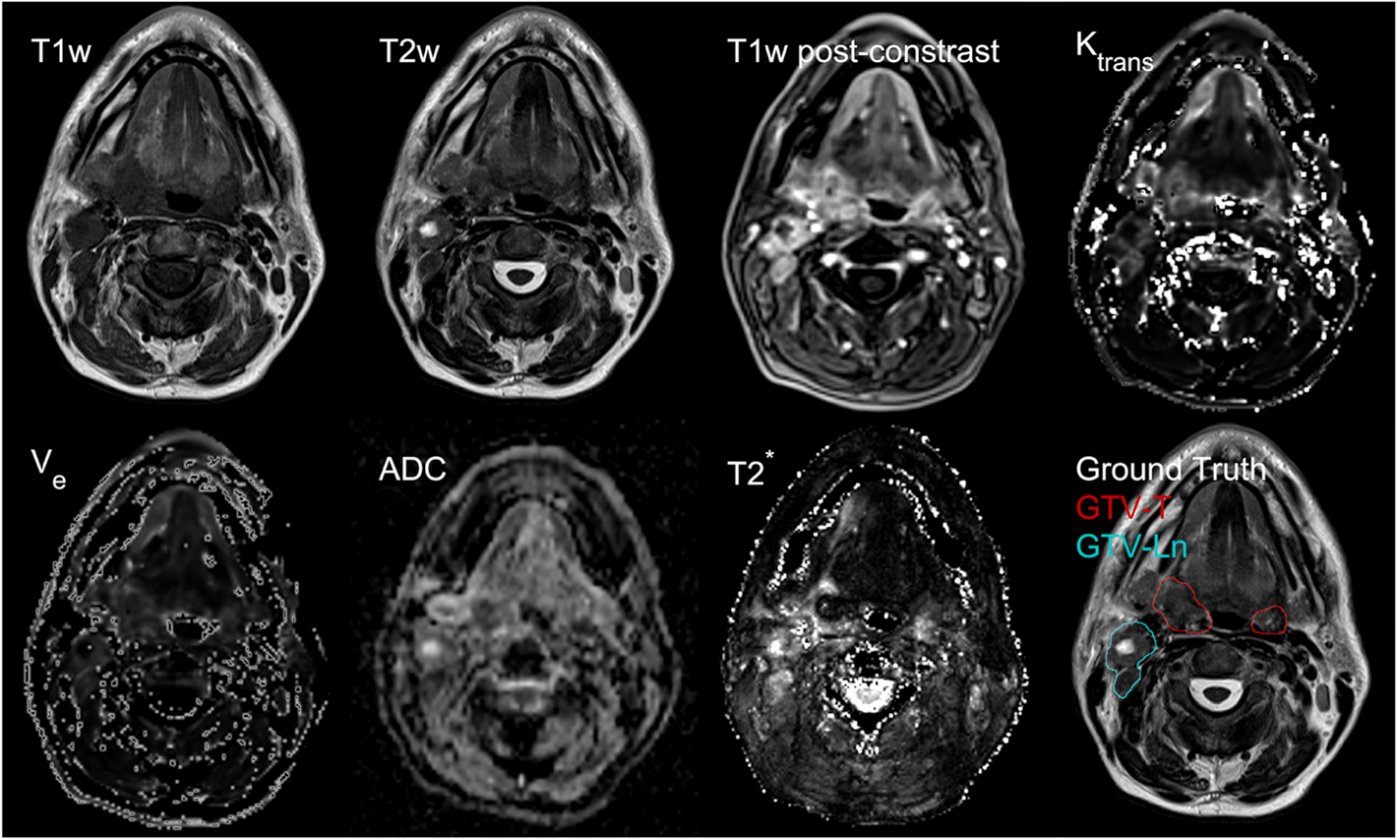
Individual co-registered slices from the 7 datasets of a head&neck tumor patient. The 7 different MRI contrasts and the ground truth GTV labels were used to train CNNs for tumor and lymph node metastasis segmentation.

For the segmentation of primary tumor and lymph node metastases, a CNN was trained with the MRI data from 7 input channels: T1w (pre-contrast), T2w, T1 (post contrast), ADC map, T2* map, and k_trans_ and v_e_ maps (Fig. 1). For the 24 patients measured at the three time points during treatment, a total of 72 datasets were available. About 50% of these data sets had to be excluded because of patient compliance, excessive motion or B_0_ inhomogeneity artifacts (especially in the diffusion sequences due to the anatomy [16]). Therefore, the CNN calculation is based on 36 complete datasets from 18 different patients, of which 13 were acquired at week 0, 9 at week 2, and 14 at week 5.

### Data pre-processing

The GTVs were exclusively contoured on the MRI data using the radiation therapy planning software iPlan (Brainlab AG, München, Germany). Contouring was done in consensus by an expert radiation oncologist and a radiologist mainly based on T1- and T2-weighted imaging sequences, with all other contrasts available for cross reference. If necessary, the data were co-registered in the same software based on a mutual information algorithm for affine transformations. To prepare the data for the CNN calculation, all MR images and GTVs were interpolated to a common base resolution of 0.45 × 0.45 × 2 mm^3^ based on the highest resolution of the available sequences present. Weighted contrasts (T1w, T2w, T1w post contrast) were normalized to a standard deviation and mean of 0.25 each. Parameter maps ADC, T2*, k_trans_ and v_e_ were normalized based on a histogram normalization approach to retain the physical and physiological meaning of the pixel values. Therefore, normalization was chosen such that across all available patients the 10 and 90 percentile of all data was scaled to the range [0, 0.5], following a similar procedure as the decile normalization in [17]. Additionally, region masks were created that excluded all regions where any one of the 7 input data channels did not contain information, e.g. due to limited field of view or extreme signal loss due to artifacts as well as air regions outside the head&neck area.

### Convolutional neural network

A CNN was set up in MATLAB (v. 2019b, MathWorks, Inc., MI, USA) based on the DeepMedic architecture [18]. We chose to use two pathways, one with original resolution and one with a factor of 3 × 3 × 1 decreased resolution in x × y × z direction. The number of convolution layers in each pathway was set to 10 with 104 feature maps each, and residual connections in layers 4, 6, 8 and 10. Kernel sizes were set to 3 × 3 × 3 for all layers except layer 1, 5 and 9 which had kernel sizes of 3 × 3 × 1 to achieve a similar size of receptive field in each physical dimension. Upsampling of the low-resolution pathway was done using a transposed convolution layer with kernel size 3 × 3 × 1 corresponding to the down sampling factor. The paths were connected by another 3 convolutional layers with 150 feature maps each and kernels sizes of 3 × 3 × 1 and two 1 × 1 × 1 fully convolutional layers. Each convolution layer was followed by a leaky ReLu activation with scale 0.01 and a dropout layer with 20% dropout. Training was employed using the Dice loss function. The input dimension of each patch was 38 × 38 × 8 and 78 × 78 × 8 (before subsampling) pixels for each path respectively.

Analogous to [18, 19], the resulting segmentation probability maps were then passed to a conditional random field for further refinement.

### Information quantification

In order to discriminate between inputs that carry a large amount of unique information for the task of automatic tumor segmentation and those that carry mostly noise we trained several CNNs based on different inputs. Therefore, we trained one network configuration with all 7 input channels (reference CNN) and 7 more in which one of the input channels was left out (leave-one-out CNN or LOO-CNN). Each network was then compared against the reference CNN in terms of segmentation performance on a separate test set. Due to the small amount of available data the size of the test cohort was reduced to a single patient and a complete leave-one-out cross validation was employed. Consequently, we trained 36 separate CNNs from scratch for each of the 8 sets of input configurations, resulting in 288 completely trained networks. In each cross validation step the training and validation cohort was kept the same across trainings on different input configurations to ensure comparability. The networks were trained in parallel on up to six AWS g4dn.xlarge instances (Amazon Web Services, Inc) equipped with a Tesla T4 GPU, launched using the MATLAB on AWS Reference Architecture.

To measure the final segmentation performance the Dice Similarity Coefficient (DSC) is used, which measures the volumetric overlap of two target volumes compared to their total volume: $\mathrm{DSC}=2\frac{\mid A\cup B\mid }{\mid A\cap B\mid }$. The DSC is scaled between 0 and 1, where a value of 0 describes no overlap at all and 1 a perfect match.

## Results

**Fig. 2:**
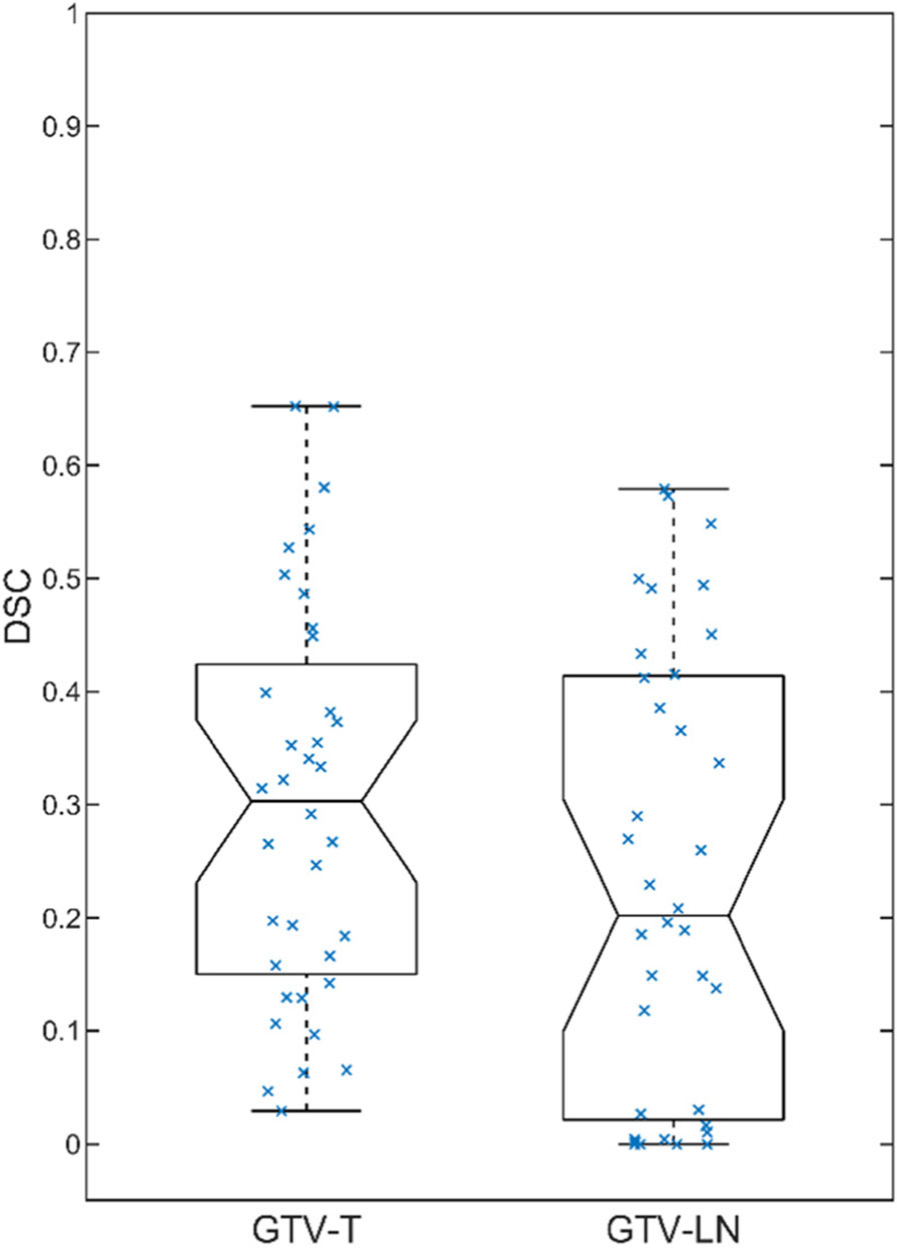
Box plot of all segmentation results on separate test sets for the reference CNN with all 7 input channels. The best segmentation performance has a DSC of 65%, GTV-T averages at 30% DSC and GTV-Ln averages at 24% DSC. The points mark all measurements and the whiskers already include all data (no outliers are drawn)

**Fig. 3:**
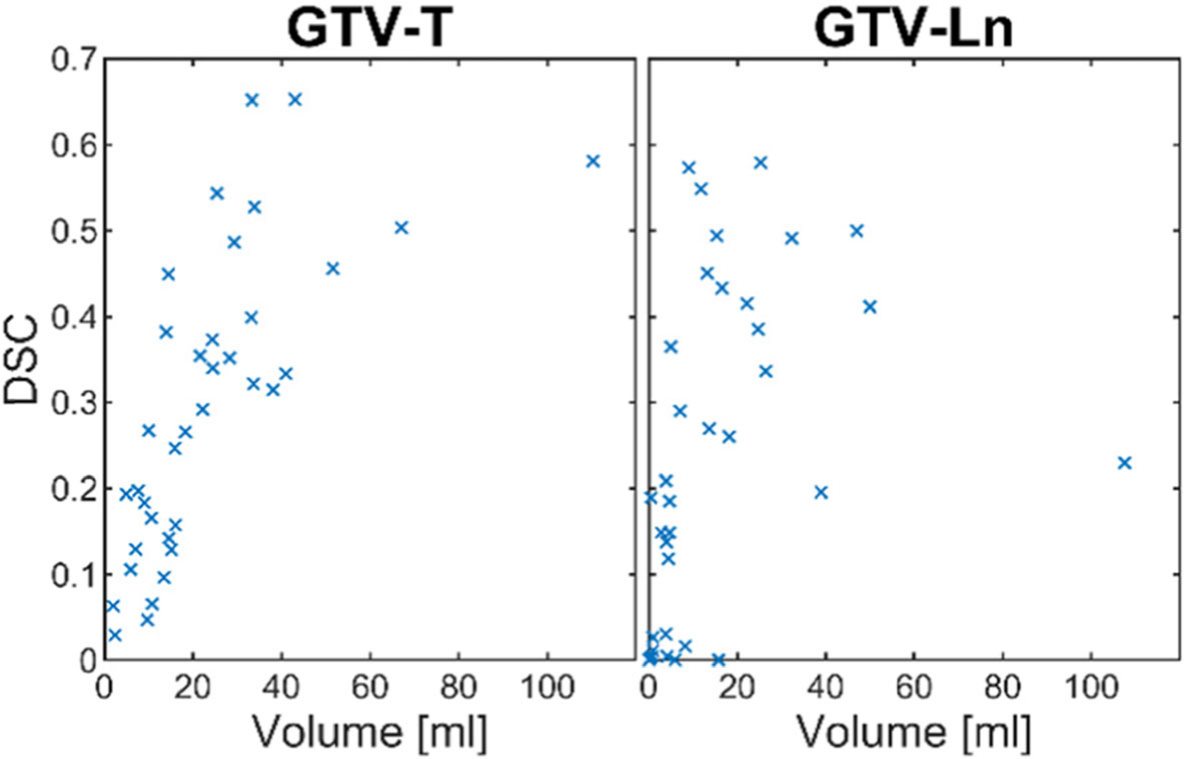
Segmentation performance plotted against the target volume. The plot shows a clear correlation between volume and segmentation performance - smaller target volumes have a lower DSC and are thus more likely to be missed, especially if the target volume is located in areas where patient movement in between imaging sequences can take place

An overall segmentation performance of up to 65% DSC in GTV-T and 58% DSC in GTV-Ln could be achieved. Figure 2 shows the complete results for the reference CNN in a box plot. Half of the test patients however showed a segmentation performance of less than 30% in GTV-T and less than 20% in GTV-LN. Comparing the segmentation performance to the lesion size shows a clear correlation as seen in Fig. 3, as with larger target volumes the segmentation generally becomes more accurate.

**Fig. 4:**
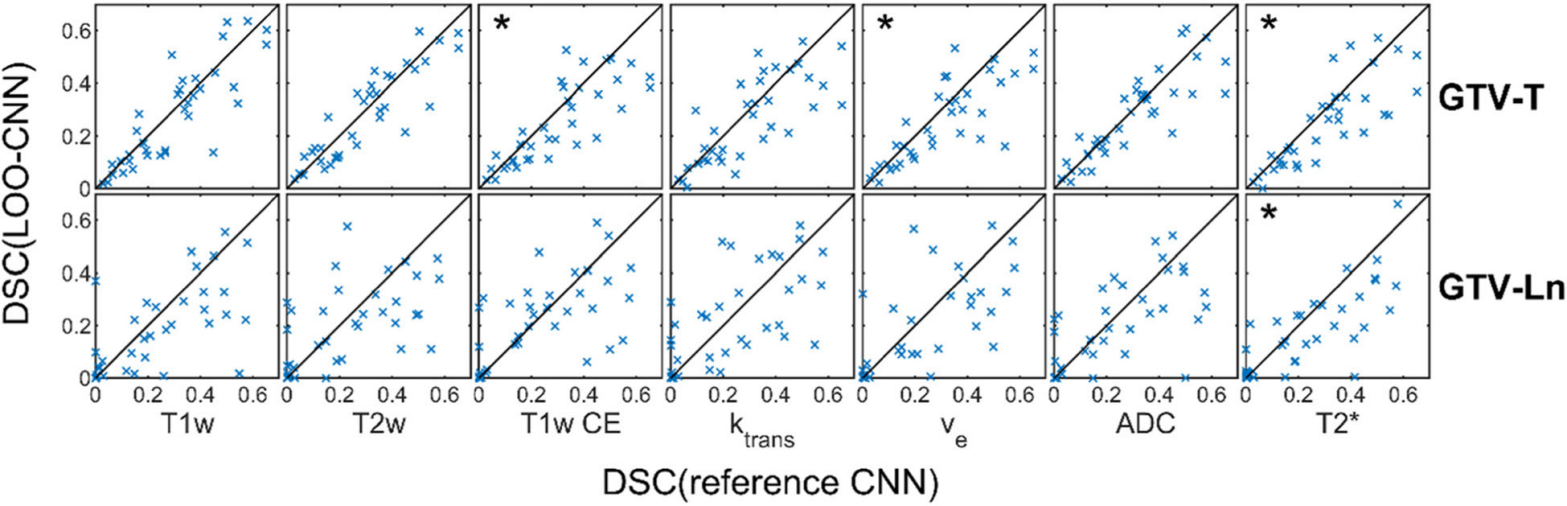
Segmentation results of the reference CNN against each LOO-CNN. The solid black line marks the line of identity. Points in the lower right mark a decreased segmentation performance compared to the reference CNN. Results that show a significant (*p* < 0.05) deviation from the line of identity are marked by an asterisk

**Fig. 5:**
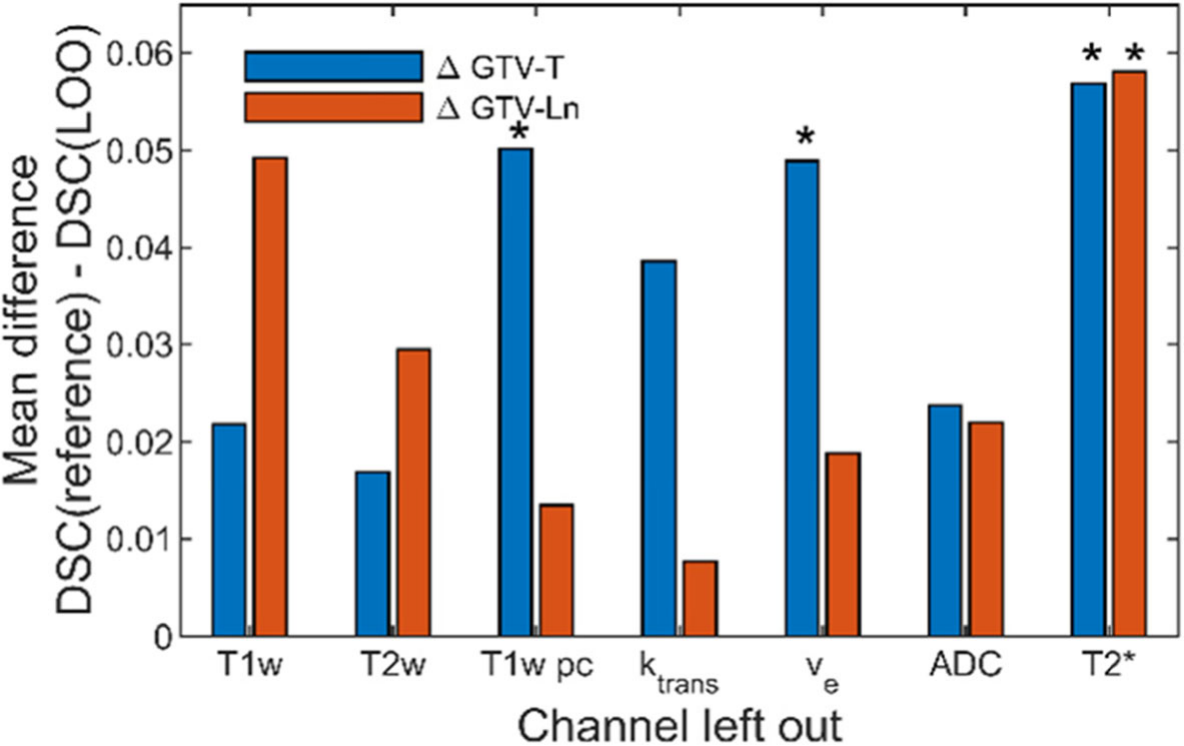
The mean DSC difference between reference CNN and each LOO-CNN shows the average decrease of segmentation performance when an individual input channel is left out. Significant differences with *p* < 0.05 are marked with an asterisk. T2* has the greatest influence on performance and ADC shows the (overall) least influence

**Fig. 6:**
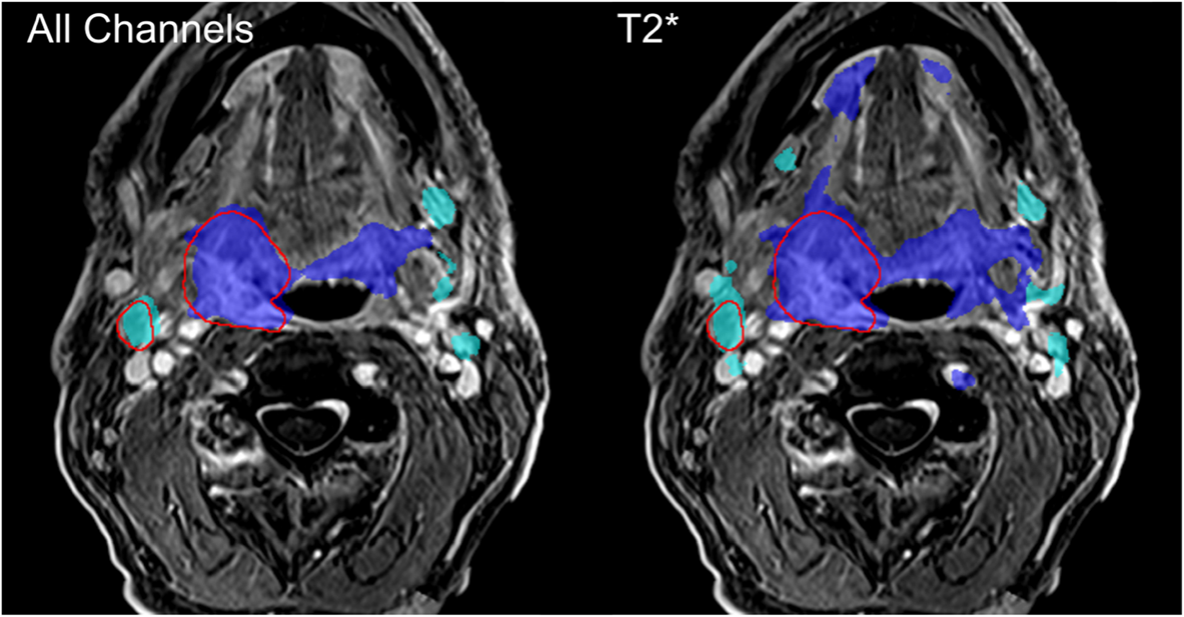
Segmentation result for the reference CNN and the LOO-CNN without T2* input. A distinct oversegmentation is observed in both cases, which is much less pronounced in the reference CNN. In this image the reference CNN has a DSC of 72% / 45% for GTV-T / GTV-Ln, while the LOO-CNN without T2* has a DSC of 56% / 39%

In Fig. 4 all test results are shown and plotted against the corresponding results of the configuration with all input channels. Paired Students-t-Tests between the segmentation results of each LOO-CNN against the results of the reference CNN showed significant differences (*p* < 0.05) for the segmentation of GTV-T without the contrast enhanced T1w, v_e_ and T2* inputs and for GTV-Ln for segmentation without T2* input. The corresponding plots are marked with an asterisk. Figure 5 shows the mean differences of the segmentation results compared to the configuration including all input channels. This comparison shows that the configuration including all inputs generally performed the best. Both significance analysis and mean difference show that the T2* contrast has the largest impact on the segmentation performance when left out. Figure 6 shows a segmentation result of a network including all input channels against one without T2* input. A distinct oversegmentation can be observed in both cases, with lesser extent in the configuration with all inputs present.

## Discussion

In this study the influence of various MRI contrasts on the segmentation performance of HNSCC with a CNN was analyzed. In particular, 8 CNNs were trained: one reference CNN with all 7 existing MRI input channels, and another 7 with each one of the channels left out (LOO-CNNs). Segmentation performance of the HNSCC GTV was then compared with the reference network to detect the image contrasts that contribute the least to the segmentation performance.

With the reference CNN a good segmentation performance was achieved with a DSC of up to 65%. A relevance analysis of the input channels was employed by comparing the LOO-CNNs to the reference CNN’s performance. For statistical robustness, mean DSC and the mean difference in DSCs (∆DSC) were used to quantify the CNN performance. In this DSC comparison it was found that the T2* contrast was the single most important input channel, both for segmentation of the tumor (ΔDSC(GTV-T) = 5.7%) and for the lymph nodes (ΔDSC(GTV-Ln) = 5.8%). The second most important contrasts were for lymph nodes the anatomical T1w (ΔDSC(GTV-Ln) = 4.9%) and for the tumor T1w CE (ΔDSC(GTV-T) = 5.0%). Perfusion parameters were the least important ones for the segmentation of GTV-Ln (0.8% k_trans_, 1.8% v_e_) but were strongly affecting GTV-T segmentation (3.9% k_trans_, 4.9% v_e_). ADC showed the least total impact on segmentation performance (2.4% on GTV-T, 2.2% on GTV-Ln). This result is surprising, as ADC has proven to be a good predictive marker in other tumor entities, e.g. for prostate or breast cancer [20–22]. However, ADC maps are often geometrically distorted due to local field inhomogeneities which can be very prominent in the neck region. As the segmentation performance measured with the DSC, which is defined by the geometrical overlap between the manually defined GTV and the segmentation, this quality measure is very sensitive to distortion.

To the best of our knowledge, this study shows for the first time the influence of 7 different input channels from MRI alone with a complete statistical evaluation on a separate test set. Through this broad spectrum of different contrasts general insight to MRI protocol optimization could be obtained. Although promising segmentation results are shown, one limitation of using 7 CNN input channels is given by the inherent non-affine deformations that cannot be avoided in the head&neck area for scanning time around 40 min. Swallowing motion and tongue movement can lead to blurring and smearing of image features in and around the tumor region so that accurate image registration between different contrasts may not always be feasible. However, previous studies could show deformable registration of distortion artifacts did not lead to an increased segmentation performance [23]. Therefore, no deformable co-registration was employed and consequently, tumor borders were not perfectly matched in every case. However, this residual motion is also present during radiotherapy and will be an unavoidable limiting factor in the precision of dose application, even though adaptive radiation therapy is able to resolve systematic and random setup errors [24].

In this study the ground truth GTVs were not always defined on the same image contrast but may have been outlined by the radiation oncologist on either the T1w post contrast or the T2w contrast – however, during planning always all images were considered for decision making. This implies that the network could not use learned borders from a single sequence in general, which is expected to decrease segmentation performance to at least the degree of the previously discussed misregistration. On the other hand, this definition of the GTVs on different contrasts can increase the robustness of the CNNs toward patient motion, as the variability of GTV definition acts as a data augmentation technique and therefore reduces the risk of overfitting [25, 26].

In other body regions segmentation performances of more than 90% have been reported [10, 18, 27, 28]. In brain tumors image coregistration is a true rigid body problem which is expected to yield better overall segmentation performance as compared to deformable registrations in the more variable head&neck region. Automatic segmentation of HNSCC on MRI data is not widely adapted yet due to missing open data access and higher degree of complexity. Automatic segmentation techniques in the head&neck area have mainly been focused on the delineation of organs at risk (OAR) and are based on CT images [10, 29, 30]. Moe et al. [31] obtained a segmentation performance of DSC = 75% for primary tumor segmentation based on PET/CT data of 197 patients. Results from a previous study on MRI images with 5 input channels [23] yielded similar DSC of 40 ± 18%, though that study was lacking a complete cross validation. The overall lower segmentation performance in this study compared to other published results can mostly be attributed to the small dataset of 36 cases. Additionally, the 36 cases do not represent the same stage of the cancer, as they are taken from 3 time points before and during primary radiochemotherapy. We assume that during treatment the tumor does not change its appearance so dramatically that learned features of the CNN would fail to classify it correctly. Therefore, to increase the number of training patients, all tumors from different time points were treated as separate and unique cases. Still, some structural changes in the tumor composition during treatment can be shown, most notably the decrease of hypoxic subvolumes during radiation therapy [32]. This is also expected to influence T2* measurements, as these are currently under consideration as a substitute marker for FMISO-PET [32–34].

Since the analysis of the relative importance of the input channels is based on the difference of the measured segmentation performances, large fluctuations (noise) and low to moderate overall performance is counterbalanced by the complete leave-one-out cross validation. The results show significant differences for T2* in the segmentation of GTV-T and GTV-Ln and significant differences for the segmentation of GTV-T for T1w post contrast and perfusion v_e_. Interestingly, a decrease in segmentation performance was seen for all networks compared to the reference network, and, therefore, each input channel contributed some unique valuable information to the segmentation network. It is expected that with a larger patient cohort the overall performance will increase and a statistically more precise assessment of the unique information contents for each input channel becomes feasible.

In future studies we will also investigate the effects of different combinations, e.g. a configuration without any anatomical or without any functional input channels. We expect that the anatomical T1w, T2w and T1w post contrast sequences include a large amount of overlapping information that can be compensated if one of those inputs is omitted. This would explain the low impact on segmentation performance if only one of these channels is left out and could be proven if all of them were omitted at the same time.

Similarly, this method can be applied to tumor segmentation in other organ regions such as prostate, breast or brain, where multi-parametric MRI data are acquired. With the rising demand of AI support systems in clinical decision making, the question of optimized imaging protocols becomes increasingly relevant. This study shows how MRI protocols can be made more time efficient, which increases patient compliance and thus, indirectly, improves image quality.

## Conclusion

In this study we demonstrated a method to quantify the information content of multiple MRI input channels in a CNN with respect to the segmentation performance of head&neck cancer. A CNN was trained on head&neck tumor and lymph node metastasis segmentation with segmentation performances up to 65% DSC. The analysis could be performed on a small dataset of 36 cases due to a statistical analysis including a thorough leave-one-out cross validation, which yielded 288 fully trained neural networks in total. Retraining the network with single input channels left out identified T2* as the single most important input channel out of 7 contrasts, and ADC as the least important to the segmentation of GTV-T and GTV-Ln combined.

## Data Availability

The datasets used and/or analyzed during the current study are available from the corresponding author on reasonable request.

## Abbreviations

AI: Artificial Intelligence
ADC: Apparent diffusion coefficient
CNN: Convolutional neural network
CT: Computed tomography
DSC: Dice Similarity Coefficient
DWI: Diffusion weighted imaging
EPI: Echo planar imaging
GTV: Gross tumor volume
GTV-T: Gross tumor volume of the primary tumor
GTV-Ln: Gross tumor volume of the lymph node metastases
HNSCC: Head and neck squamous cell carcinoma
LOO-CNN: Leave-One-Out CNN, which is a CNN with one of the input channels left out
MRI: Magnetic resonance imaging
NN: Neural network
PET: Positron emission tomography
TA: Acquisition time
TE: Echo time
TR: Repetition time
T1w: T1-weighted
T2w: T2-weighted
